# A comparative study of transabdominal and transvaginal ultrasound guidance on consequences of embryo transfer at Mahdiyeh hospital of Tehran in 2020: An RCT

**DOI:** 10.18502/ijrm.v20i3.10708

**Published:** 2022-04-21

**Authors:** Paria Geran Malekkheili, Shahrzad Zadehmodarres, Zahra Heidar

**Affiliations:** Clinical Research Development Center, Mahdiyeh Educational Hospital, Shahid Beheshti University of Medical Sciences, Tehran, Iran.

**Keywords:** Transvaginal, Transabdominal, Ultrasound, Embryo transfer, Pregnancy.

## Abstract

**Background:**

Infertility is an important problem that affects many couples worldwide. Assisted reproductive technology (ART) helps infertile couples to have offspring. One of the critical parts of ART is embryo transfer (ET).

**Objective:**

To compare the effect of transvaginal and transabdominal ultrasonography-guided ET on ART outcomes.

**Materials and Methods:**

In this randomized clinical trial study, 90 women who were candidates for in vitro fertilization (IVF) referred to Mahdiyeh hospital of Tehran, Iran during the yr 2020 were randomly divided into two groups (n = 45/each) of transvaginal and transabdominal ultrasonography-guided ET. The embryos were transferred two-three days after oocyte retrieval. The patient pain, duration and difficulty of the procedure, three-dimensional vision quality and successful pregnancy rate were measured.

**Results:**

In this study, 63.2% of the 45 women who underwent IVF under the guidance of the transvaginal guidance and 36.8% of the 45 women who underwent IVF under the transabdominal guidance had a successful pregnancy, which was not significantly different (p = 0.19). Also, based on other results there was no difference between the two groups in terms of patient pain (p = 0.53), duration (p ≥ 0.50), difficulty of procedures (p ≥ 0.50) and ultrasonography vision; however, the three-dimensional vision quality in the transvaginal ultrasonography was better than that in the transabdominal ultrasonography (p < 0.01).

**Conclusion:**

Overall, the ART outcomes in the transvaginal and transabdominal ultrasonography-guided ET were similar, so we suggest that physicians evaluate the patient's situation, the hospital equipment, and their ability before selecting the type of ultrasonography.

## 1. Introduction

Infertility affects the life of nearly 15% of couples worldwide (1). It occurs due to male, female, combined, and idiopathic problems. Some techniques give infertile couples a chance to have healthy offspring, such as intrauterine insemination, in vitro fertilization (IVF), intracytoplasmic sperm injection, etc. (2). The success rate of these techniques depends on different factors such as the ovulation-induction protocols (3), the culture media (4), the women's age (5), the technique of embryo transfer (ET) (6), etc. ET is one of the critical parts of assisted reproductive technology (ART) and it affects ART outcomes. ET depends on many factors such as the skills of the operator or specialist (7), the type of catheter (8), the part of the uterus where the embryos are discharged (9), the time of the interval between the embryo catheter loading and the embryos discharging (10), blood or mucus contamination (11), contraction of the uterus, the level of difficulty in the catheter passing through the cervix (12), ultrasound guidance (6, 13), and pain (14).

The tip of the catheter should not touch the fundus of the uterus. If this happens, the patient experiences suprapubic pain and pressure, which may be a consequence of uterine contraction. Contraction of the uterus at the time of ET decreases the clinical pregnancy rate (14). The cause of the pain is blindly inserting the catheter and discharging the embryos. Ultrasound-guided fetal transfer helps appropriate transfer and prevents it from reaching the uterine fundus. This allows the specialist to see the transferic point immediately after the fetus is emptied and to follow the position of the fetus in the uterus (15). In the past, the position and condition of the uterus were examined by clinical touch just before ET (16); however, now the uterus is evaluated with ultrasonography. This gives specialists valuable information about the size and position of the uterus, the length and angulation of the cervical canal, and the fibroids in the uterus cavity, all of which is very helpful for guiding the specialist at the time of ET (17). Therefore, according to several recent studies, the use of ultrasonography guidance improves ART outcomes (18, 19). However, the major limitation of ultrasonography-guided ET is that it is time-consuming, which leads to the patient having a full bladder (19).

At first, transabdominal sonography was done at the time of ET. In 1990, for the first time, transvaginal sonography was performed to do an ET (20, 21). A few problems were encountered, such as patient's discomfort because of full bladder and anxiety for emptying the bladder immediately after ET, and the possibility of not seeing the ET catheter during the procedure in obese patients or patients with uterus abnormality (22, 23).

Therefore, considering the effect of ultrasonography guidance at the time of ET on ART outcomes, the aim of the present study was to compare the efficacy of using transvaginal and transabdominal sonographies during ET.

## 2. Materials and Methods 

### Study characteristics and patient inclusion and exclusion criteria

This clinical trial research was performed during a period of 10 days in 2020 at Mahdiyeh hospital of Tehran, Iran. In this study, 147 women were recruited, of which 49 were excluded for failing to meet the inclusion criteria and eight due to unwillingness to participate in the study. Finally, 90 women who were IVF candidates entered the study. According to the normal range of Anti-Müllerian hormone (AMH), the patients with AMH > 0.7 were included. Also, antral follicle count and AMH were always used together to determine ovary response and so patients with poor responses were not included in this study. The inclusion criteria were women aged 18-40 yr old who were candidates for IVF. Women with the body mass index (BMI) > 38 kg/m^2^, uterus anatomical malformation, and dissatisfaction were excluded.

The women were randomly divided by using the simple randomization method, and the individual unit and division of people was done using the Research Randomizer software, version 3.0 and random allocation with a ratio of 1: 1 into two groups: transvaginal ultrasonography-guided ET and transabdominal ultrasonography-guided ET. A Honda device made in China with H-S2000 probe was used for ultrasound.

### Ovulation stimulation, endometrial preparation, and luteal phase support protocols

Long or flare protocols or antagonists can be used to stimulate the ovaries. In this study, all patients underwent IVF according to the antagonist protocol. From the third day of their cycle, at least 75-150 units of Gonal-F (Merck, Germany) were injected into patients subcutaneously daily until the day of the puncture. Then, on the sixth day, sonography was performed and the dose of Gonal-F was adjusted. After observing 12-13 mm follicles, Cetrorelix (Merck Serono, Switzerland) was administered daily subcutaneously at a dose of 0.025 mg until the day of puncture. Three-four days later, another sonography was performed. Menopur was used if a luteinizing hormone surge was needed. This stimulation continued until at least three-five follicles sized 16-20 mm were found in the transvaginal ultrasound. Then, in patients with a BMI < 30, one 500 mg Ovitrell ampoule (Merk, UK) was administered and in patients with a BMI > 30, two 500 mg Ovitrell ampoules (Merk, UK) were administered. Follicles > 10 mm were aspired 36 hr later under sedation by propofol (Sigmak Lifesciences, India) and guided by transvaginal sonography (Honda, China). In the case of symptoms of hyperstimulation in the patient, 0.2 units of Diphereline (NPS Medicine Wise, Australia) was used instead of Ovitrell. In the event of an increased response, the embryos were usually frozen and the transfer of the embryo to the next cycle was delayed. Until fertilization, two 50 mg ampoules of progesterone (Pfizer CentreOne, Germany) were administered daily. Semen samples were taken immediately before oocyte recycling and fertilized the oocytes by using the intracytoplasmic sperm injection method. The embryos were then incubated for 16-18 hr in an incubator (Memmert, Germany) with 99% humidity, at 37ºC and 50% CO ${}_{2}$ . Then, they were examined under a microscope (Optika, Italy) and if pronucleus of the male and female were seen, the embryos were kept in a special culture medium for three-five days until the transfer and reaching the eight-cell stage. Grade A and B embryos were usually transferred for one-three embryos depending on the age of the patient. Generally, three embryos were transferred in women aged > 35 yr and one-two embryos in those aged < 35 yr. Enoxaparin (40 mg, Lovenox, Singapore) was used for up to 2 wk after ET and determination of β-human-derived chorionic gonadotrophic titer. It was noted that at this time the thickness of the endometrium was about 6-7 cm. To support the luteal phase, a 400 mg Cyclogest (Actover, Iran) suppository was used.

### ET technique and ultrasonography

ET was performed two-three days after oocyte retrieval. In both groups, the vagina was filled with 10 ccs of lubricant gel (Kodex, Iran), and based on the specialist's decision, two catheters (SIVF, CooperSurgical, Germany or Edwards-Wallace, CooperSurgical, Germany) were used. In the transabdominal ultrasonography-guided ET group, the patient's bladder was full. The ultrasonography device was a Honda machine made in China; the vaginal probe was H-S2100 and the abdominal probe was H-S2000.

### Assessments

The patients and the specialists were asked about the patient's pain, the procedure duration and difficulty, three-dimensional (3D) vision quality and frequency of tenaculumn. Finally the rate of successful pregnancy was calculated in both groups.

The patient's pain was measured during the ultrasound and at the end of the ultrasound. The procedure duration and difficulty, 3D vision quality and frequency of tenaculumn were measured at the end of the ultrasound. The rate of successful pregnancy was measured four wk after fetus transfer.

### Ethical considerations

This research was approved by the Ethics Committee of Shahid Beheshti University of Medical Sciences, Tehran, Iran (Code: IR.SBMU.RETECH.REC.1397.870). All participants were completely aware of the study conditions and gave written consent prior to the study.

### Statistical analysis

For the statistical analysis of the data, both *t* test and Chi-square test were performed using the Statistical Package for Social Sciences software, version 18.0 (SPSS, SPSS Inc., USA). The significance level was considered as p-value < 0.05.

## 3. Results

**Figure 1:**
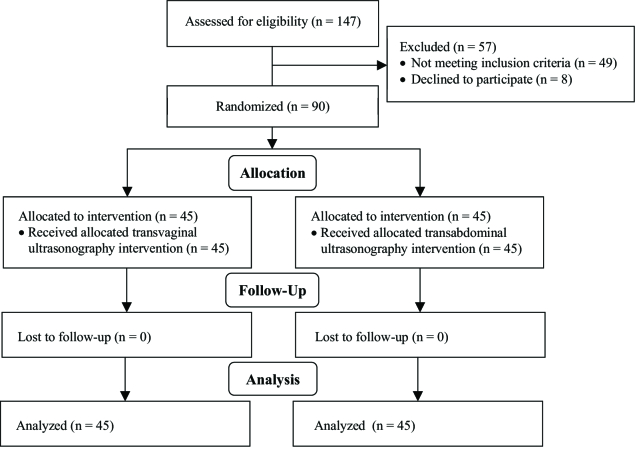
Consort diagram of the study.

In this study, 147 women participated in the first phase. Of those, 49 failed to meet the inclusion criteria and eight women were unwilling to participate and therefore were excluded. Finally, 90 women entered the main phase of the study (Figure 1).

Table I demonstrates the frequency of tenaculum uses and successful pregnancy and 3D vision quality. The frequency of tenaculum uses in both the transvaginal and transabdominal ultrasonography-guided ET were six (p = 1.00). Hence, no difference was noted between the two groups in the frequency of the tenaculum use, so the type of sonography had no effect on the use of tenaculum. Successful pregnancy after transvaginal ultrasonography-guided ET was 12 and after transabdominal ultrasonography-guided ET was seven. The p-value was 0.19, so there was no significant difference between the two groups and the type of ultrasonography had no effect on the successful pregnancy rate. Table II shows the 3D vision quality for the transvaginal and transabdominal ultrasonography. The p-value was < 0.01, so the 3D vision during the transvaginal ultrasonography was significantly better than during the transabdominal ultrasonography.

As shown in table III, the mean number of transferred embryos in transvaginal ultrasonography-guided ET was 2.58 and in transabdominal ultrasonography-guided ET was 2.53 with a p-value of 0.80, so there was no significant difference between the two groups.

Also, the mean of the pain measurement of the patients is demonstrated in table III. The p-value was 0.53, so the method of ultrasonography had no effect on pain.

Table IV presents the results of the duration of the procedure demonstrating that most procedures lasted < five min (p < 0.01) and there was no significant difference between the two groups.

Table V presents the difficulty of the procedure, as reported by the specialists: there was no significant difference in the difficulty of the procedures between the two groups (p = 0.50).

Lubricant gel or saline was only used to reduce pain when placing the speculum. Also, during the transvaginal ultrasound, the gel was put directly on the vaginal probe and then sterile gloves were placed on the probe and inserted into the vagina.

**Table 1:** Demographic information of patients


**Demographic information**		**Frequency (percentage)**	**P-value**
**Age (yr)**			
	**18-25**	6 (6.66)	0.57
	**26-30**	15 (16.66)	
	**31-35**	36 (40.00)	
	**36-40**	33 (36.66)	
**Laparoscopic history**			
	**Positive**	27 (30)	0.51
	**Negative**	63 (70)	
**BMI (kg/m^2^)**			
	**20-25**	18 (20.00)	0.53
	**26-30**	48 (53.33)	
	**31-35**	24 (26.66)	
**Infertility**			
	**Primary**	75 (83.33)	0.5
	**Secondary**	15 (16.66)	
Data were analyzed by both *t* test and Chi-square test. BMI: Body mass index			

**Table 2:** Frequency of tenaculum use and successful pregnancy, and quality of the 3D vision


**Groups**	**Transvaginal ultrasonography**	**Transabdominal ultrasonography**	**Total**
**No use of tenaculum**	39 (50.0)	39 (50.0)	78 (100)
**Use of tenaculum**	6 (50.0)	6 (50.0)	12 (100)
**Unsuccessful pregnancy**	33 (46.5)	38 (53.5)	71 (100)
**Successful pregnancy**	12 (63.2)	7 (36.8)	19 (100)
**Poor 3D vision**	1 (14.3)	6 (85.7)	7 (100)
**Good 3D vison**	23 (42.6)	31 (54.4)	54 (100)
**Excellent 3D vision**	21 (72.4)	8 (27.6)	29 (100)
Data presented as n (%). Data were analyzed by both *t* test and Chi-square test. P-values of tenaculum use, successful pregnancy, and 3D vision were p = 1.00, p = 0.19, and p < 0.01, respectively			

**Table 3:** The mean number of transferred embryos and pain comparison test at the time of ET


**Groups**	**Transvaginal ultrasonography**	**Transabdominal ultrasonography**
**Mean number of transferred embryos**	2.58 ± 0.78	2.53 ± 0.89
**P-value of transferred embryos**	0.80	0.80
**Mean of pain comparison test**	2.49 ± 1.14	2.62 ± 0.88
**P-value of pain comparison test**	0.53	0.53
Data were analyzed by both *t* test and Chi-square test		

**Table 4:** Duration of procedure


**Groups**	**Transvaginal ultrasonography**	**Transabdominal ultrasonography**	**Total**
**0-5 min (percentage %)**	42 (48.3)	45 (51.7)	87 (100)
**5-10 min (percentage %)**	3 (100)	0 (0)	3 (100)
**P-value of procedure duration**	0.50	0.50	
Data were analyzed by both *t* test and Chi-square test			

**Table 5:** Difficulty of procedure


**Groups**		**Frequency (percentage %)**
**Transvaginal ultrasonography**		
	**Easy**	25 (56.6)
	**Moderate**	20 (44.4)
	**Difficult**	0 (0)
** Transabdominal ultrasonography**		
	**Easy**	20 (44.4)
	**Moderate**	24 (53.3)
	**Difficult**	1 (2.2)
**Total**		90 (100)
Data were analyzed by both *t* test and Chi-square test. P-value of procedure difficulty was p = 0.50		

## 4. Discussion

Infertility affects many couples worldwide (1), and the use of ART helps many of them by giving them a chance to have healthy offspring. Many factors influence the outcome of ART; ET in the clinical stage has the most influence on outcome (6). In the present study, we evaluated the effect of two main methods of ultrasonography at the time of ET on patient's pain, the comfort of patients and specialists, and the successful pregnancy rate.

The transvaginal ultrasonography-guided ET was not associated with a lower frequency of tenaculum use in comparison with transabdominal ultrasonography-guided ET. It also did not appear to impact the duration or difficulty of the procedure. The mean number of transferred embryos in each patient was equal in the two groups; therefore, matching between the two groups was well done. Despite the better 3D vision in transvaginal ultrasonography compared to transabdominal ultrasonography, the successful pregnancy rate and pain were not different between the two groups. Previous studies have demonstrated that the use of ultrasonography at the time of ET can improve the outcomes of ART such as the implantation and pregnancy rates compared to ET after just a clinical touch; it has been suggested that this improvement is because of the better evaluation of the uterus for any anatomical malformations and position, and of the area of embryo discharging. Ultrasonography has also been found to decrease the rate of ectopic pregnancy (16, 18, 19, 24). There was no significant difference between the two groups in the prevalence of successful pregnancy. The use of transvaginal and transabdominal sonography is a challenge. Some studies have found that transvaginal ultrasonography can improve the pregnancy rate in patients who have had failed IVF cycles (22) and that the use of transvaginal ultrasonography can increase the prevalence of pregnancy and implantation rates (6). In other studies, transvaginal ultrasonography reduced the pain and discomfort of patients due to a full bladder so the patients had a better emotional state at the time of ET (25, 26). Many studies have illustrated that transvaginal ultrasonography-guided ET and transabdominal ultrasonography-guided ET have similar effects on clinical pregnancy, implantation rate (26), live-birth rates, the time required for ET (27), and transfer difficulty (25), which is also confirmed by our data.

Based on our results, the pain in both groups was similar and the successful pregnancy rate was similar too. Another study evaluated the relation between pain and ART outcome which indicated that less pain during ET is associated with a higher chance of clinical pregnancy (14); given that in our research the pain was similar, it had no effect on the successful pregnancy rate.

The duration of the transvaginal ultrasonography process was longer than the transabdominal ultrasonography-guided ET but this difference was not significant, unlike other studies which have reported that the duration of transvaginal ultrasonography-guided ET is longer (25, 26). Moreover, transvaginal ultrasonography induced more patient comfort and better endometrial visualization, which has been confirmed by another study (24). So, the specialist should consider the patients' and their own comfort while choosing the better approach.

## 5. Conclusion

Given that the transvaginal and transabdominal ultrasonography-guided ET had approximately similar effects on ART outcomes and comfort of patients and specialists, we suggest that specialists make a decision based on the patient's situation, the hospital equipment, and the specialist's abilities.

##  Conflict of Interest 

The authors declare that there is no conflict of interest.
